# G protein-coupled receptors in prostate cancer: research progress and therapeutic targets

**DOI:** 10.3389/fcell.2025.1709098

**Published:** 2025-11-19

**Authors:** Wei Xiong, Bingpeng Zhou, Jian Shi, Dong Ni, Zhiyong Xiong

**Affiliations:** 1 Department of Nephrology, Union Hospital, Tongji Medical College, Huazhong University of Science and Technology, Wuhan, China; 2 Department of Urology, Qinghai Provincial People’s Hospital, Xining, Qinghai, China; 3 Department of Urology, Union Hospital, Tongji Medical College, Huazhong University of Science and Technology, Wuhan, China

**Keywords:** prostate cancer, G protein-coupled receptors, orphan receptors, chemokine receptors, hormone-responsive GPCRs

## Abstract

Prostate cancer (PCa) is one of the most common cancers of male genitourinary system, with castration-resistant prostate cancer (CRPC) posing a major therapeutic challenge. G protein-coupled receptors (GPCRs), the largest family of cell surface receptors, are increasingly recognized as critical regulators of tumor progression, metastasis, and therapy resistance. This review summarizes the research progress of orphan receptors, chemokine receptors and hormone-sensitive receptors of GPCRs in PCa. We highlight how these receptors modulate key oncogenic processes such as androgen receptor (AR) signaling, cell proliferation, migration, and immune evasion. Emerging therapeutic strategies targeting GPCRs, including biased ligands and combination therapies, are discussed. This synthesis provides a mechanistic foundation for understanding GPCR functions in PCa and identifies promising directions for future research and drug development.

## 1 Introduction

Prostate cancer (PCa) is a highly prevalent malignant tumor among men globally, with over 1.2 million new cases diagnosed each year and more than 350,000 deaths related to the disease (2021). Although the majority of cases have a slow progression and pose no threat to the mortality rate, many patients present with moderate or high-risk localized, locally advanced, or metastatic cancers, and still succumb to the disease despite receiving treatment (Teo et al., 2019). Thus, prostate cancer is the third leading cause of cancer-related deaths in American men.

Prostate cancer displays androgen dependence, requiring dihydrotestosterone (DHT) for growth (Molina and Belldegrun, 2011). Hormonal therapy targeting androgen signaling is the preferred treatment approach, primarily aimed at reducing androgen levels to impair tumor growth (Litwin and Tan, 2017; Fontana et al., 2020). However, a major limitation of hormone therapy is that it only provides temporary relief, with castration resistance eventually recurring in the majority of patients within 14–20 months (Sharifi et al., 2005). Despite high testosterone levels, prostate-specific antigen (PSA) levels often rise, indicating the inappropriate restoration of the AR signaling axis remains a key driver of this progressive and lethal disease (Scher and Sawyers, 2005; Ryan and Tindall, 2011). This occurs due to the development of resistance mechanisms, including amplification or mutation of the androgen receptor (AR) gene (Ruizeveld de Winter et al., 1994; Taplin et al., 2003), increased expression of AR splice variants (Sun et al., 2010), and activation of alternative signaling pathways that regulate tumor growth independently of androgens.

**FIGURE 1:**
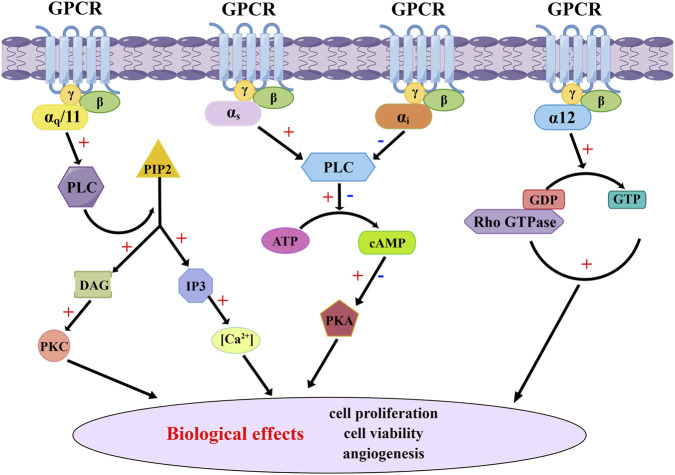
G proteins-mediated GPCR canonical signaling pathway. The canonical signaling pathway of GPCR mediated by G proteins involves a conformational change of G proteins and subsequent release of GDP upon activation by agonists. This event facilitates the rapid binding of GTP to G proteins, resulting in the dissociation of Gα subunits from both the receptor and Gβγ subunits. Active Gα subunits further activate various intracellular signaling pathways, including the adenylyl cyclase pathway and changes in intracellular Ca^2+^ concentration, leading to diverse cellular biological effects.

**FIGURE 2:**
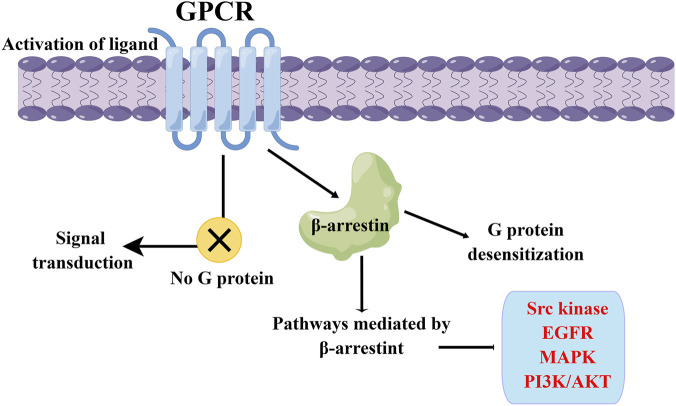
β-arrestin-mediated GPCR biased signaling pathway. The β-arrestin signaling pathway can induce desensitization of G proteins, and it can also mediate the transmission of various signaling pathways, including Src kinase, EGFR, MAPK, and PI3K/Akt.

The G protein-coupled receptors (GPCRs) represent the largest family of cell surface receptors in the human genome, with over 900 members, and they control the majority of physiological functions through G protein signaling (Pierce et al., 2002; Kobilka, 2007). GPCRs consists of a peptide chain containing seven alpha-helical transmembrane domains that divide the receptor into extracellular N-terminal, three extracellular rings, intracellular C-terminal, and three intracellular rings. After binding with excitatory signaling molecules (such as odor, hormone, neurotransmitter and chemokine), conformational changes occur in the extracellular region of GPCRs, which then trigger the movement of transmembrane helices, especially the intracellular half of TM6 that is close to the intracellular helices. At this time, the intracellular region that activates the receptor can recruit and bind downstream effector proteins (such as G protein, B-arrestin, etc.), which regulate physiological activities *in vivo* through cyclic adenylate (cAMP) signaling pathway, phosphatidylinositol signaling pathway and calcium ion signaling pathway (Hauser et al., 2018). Figure 1 illustrates the canonical signaling pathway of GPCRs mediated by G proteins, while Figure 2 depicts the biased signaling pathway of GPCRs mediated by β-arrestin. GPCRs can activate a wide range of physiological responses, such as neurotransmission, hormone and enzyme release from endocrine and exocrine glands, immune reactions, cardiac and smooth muscle contraction, and blood pressure regulation, by modulating the activity of intracellular signaling pathways through G proteins and β-arrestins (Gurevich and Gurevich, 2019). The importance of GPCRs makes it one of the most important families of drug targets, playing a vital role in drug discovery for major diseases such as cardiovascular disease, metabolic diseases, neuro-related diseases, immune diseases, and cancer. Currently, there are about 500 GPCRs-targeting drugs, accounting for 34% of FDA approved (Kenakin, 2012; Hauser et al., 2017; Hauser et al., 2018). Programmed cell death protein 1 (PD1) or programmed death-ligand 1 (PD-L1) and cytotoxic T-lymphocyte associated protein 4 (CTLA4) inhibitors combined with small molecule drugs targeting GPCRs for the treatment of cancer are now in clinical trials (He et al., 2022).

Emerging experimental and clinical data have demonstrated that GPCRs regulate processes such as proliferation signal transduction, cell cycle, apoptosis, growth inhibitory factors, angiogenesis, and cell proliferation and migration, which play critical roles in cancer development and progression (O'Hayre et al., 2014). Increasing evidence suggests that GPCRs, G proteins, and their downstream signaling targets are involved in regulating the maintenance, differentiation, and multipotency of cancer stem cells (Chaudhary and Kim, 2021). Aberrant overexpression of GPCRs and their activation by autocrine and paracrine agonists released by tumor or stromal cells represent the most common strategy employed by cancer cells to stimulate GPCR signaling networks (Bar-Shavit et al., 2016). Currently, drugs targeting GPCRs have shown excellent therapeutic efficacy, owing to their ability to target GPCRs, like many other types of cell surface proteins, in several malignancies (Soond and Zamyatnin, 2020; Bair et al., 2024). Understanding the mechanistic relationship between GPCRs and malignant tumors is crucial, as they represent feasible targets for cancer treatment development.

There is increasing research interest in exploring the molecular and related mechanisms involved in the regulation of prostate cancer that are independent of androgen and AR. GPCRs, which are membrane proteins that play a crucial role in cell signaling pathways, participate in the regulation of various cellular processes in prostate cancer, including cell proliferation, migration, and apoptosis (Rodriguez et al., 2016a; Luo et al., 2017; Heidegger et al., 2022; Hou et al., 2025; Zhang T. et al., 2025). Furthermore, GPCRs can serve as targets for developing novel therapeutic strategies for prostate cancer. In the following sections, we will review the specific mechanisms by which GPCRs regulate the development of prostate cancer, as well as potential therapeutic strategies targeting these receptors.

## 2 Orphan receptors in prostate cancer

Orphan receptors refer to some receptors which are obviously similar to other recognized receptors in structure, but whose endogenous ligands have not been found. The identification of novel receptors with structures similar to existing receptors has been increasing rapidly (Giguere, 1999). If the endogenous ligand has not been identified, the receptor is called an “orphan”. If a ligand is later found, the receptor is said to be “adopted orphan” (Nanduri et al., 2015). Orphan receptors in prostate cancer are mainly divided into two categories, the first are GPCRs and the second are nuclear receptors. Nearly 100 receptor-like genes in the GPCR family are still orphans (Trzaskowski et al., 2012).

### 2.1 Olfactory receptors

The olfactory receptor (OR) gene family represents the largest subgroup of GPCRs. *OR51E2*, also known as prostate-specific G protein-coupled receptor 1 (PSGR1), is one of the most studied ORs in PCa (Xu et al., 2022). Most studies on PSGR1 have focused on its pathophysiological functions, which suggest that it serves as a biomarker for prostate cancer and is involved in the development and progression of the disease. *OR51E2*-knockout cells showed increased proliferation, migration, adhesion, colony formation, and tumor growth, leading to a more aggressive cancer phenotype. TCGA cohort analysis showed that prostate cancer patients with low tumor *OR51E2* expression had a worse prognosis and a higher average Gleason grade than those with higher expression (Thomsen et al., 2025).

It is widely accepted that PSGR1 promotes the progression of prostate cancer by activating NF-κB to regulate inflammation-related factors. Rodriguez et al. demonstrated that PSGR1 activated NF-κB via the AKT pathway to induce a pro-inflammatory phenotype in the prostate, leading to low-grade prostatic intraepithelial neoplasia (PIN) (Rodriguez et al., 2014). Studies by Melissa et al. suggested that overexpression of PSGR1 not only reduced the expression of AR in prostate epithelial cells but also increased the expression of AR in the prostate stroma, thereby promoting cell proliferation induction. Furthermore, overexpression of PSGR1 itself could activate NF-κB, inducing the proliferation of prostate epithelial cells. In addition, elevated expression of PSGR1, in combination with *PTEN* loss, promoted the progression of prostate cancer (Stone, 2015; Rodriguez et al., 2016b). Some other studies believe that MAPK pathway is also an important pathway for PSGR1 to play a role in prostate cancer, although its exact role appears context-dependent. Xin and colleagues proposed that the hyperactivation of the oncogenic MAPK pathway in prostate cancer was partly due to PSGR1-induced activation of MAPKs ERK1/2 via a specific GA-localized Gβγ-PI3Kγ-ARF1 pathway (Xu et al., 2022). In contrast, another study found that the ligand β-ionone activates PSGR1, which led to the activation of the p38 and JNK signaling pathways in the MAPK family, inhibiting AR transactivation and suppressing PCa cell growth (Xie et al., 2019). This discrepancy suggests that PSGR1’s engagement with the MAPK cascade and the resultant cellular effects may be ligand-specific and/or pathway-biased, warranting further investigation. In addition to the growth and progression of prostate cancer itself, research has been proposed that exosome PSGR1 might regulate MAPK and NF-κB signaling pathways involved in prostate cancer bone metastasis by targeting ICAM1, RELB, and IL1B (Li et al., 2020).

PSGR2, belonging to the OR receptor family and also known as *OR51E1*, is a novel human prostate-specific G protein-coupled receptor gene that is overexpressed in human PCa (Weng et al., 2006). PSGR2 is primarily located in the apical cytoplasm but also found in the basal glandular structures. Massberg et al. obtained RNA-Seq data by high-throughput sequencing, and verified the abnormal expression of PSGR2 in prostate cancer tissues by RT-PCR, which was the highest in benign prostate tissues and almost undetectable in prostate cancer tissues, all lymph nodes and distant metastatic specimens. It can be concluded that PSGR2 plays an important role in advanced prostate cancer (Maßberg et al., 2016). PSGR2’s expression is restricted to prostate tissue, giving it higher tissue specificity compared to PSA as a diagnostic marker for prostate tumors. Studies by Alexey et al. have shown that PSGR2 mediates the activation of adenylate cyclase by fatty acids, and cAMP response to these ligands only occurs in the presence of PSGR2 (Pronin and Slepak, 2021). In addition, Désirée et al. have demonstrated that the PSGR2 agonist, nonanoic acid (NA), induces growth arrest of androgen-sensitive LNCaP cells through protein kinase and various cell cycle regulatory factor pathways (Maßberg et al., 2016). Related research indicates that activation of PSGR2 inhibits cell proliferation and induces cellular senescence in PCa cells, during which PSGR2 can promote cell death by increasing ERK1/2 phosphorylation and upregulating p53 (Sakellakis, 2022).

In conclusion, *OR51E1, OR51E2* or other olfactory receptors play an important role in the genesis, growth and treatment of prostate cancer cells, but their mechanisms need further study.

### 2.2 Taste receptors

Taste receptors, first discovered in the tongue, facilitate the detection of chemicals. Notably, mammalian bitter, sweet, and umami receptors are GPCRs. In addition to the taste cells in the taste buds, the tissue cells distributed in the respiratory tract, brain, pancreas and intestine can also express taste receptors. These cells belong to extragustatory chemosensory cells (Lee and Cohen, 2015), which are not coupled with neurons. It does not transmit taste signals to the central nervous system, but plays an important role in resisting microbial infection, regulating nutrient absorption and maintaining homeostasis of the internal environment, which can have an important influence on the development of related tissues and organs and organism diseases.

Bitter receptors are reported to be expressed in cells, tissues, and organs of the genitourinary system (GU), and taste receptors may signal downstream to activate an inflammatory cascade, suggesting that these receptors may play an integral role in mediating the inflammatory response to microbial invasion of the GU. Bitter taste receptors (TAS2Rs) have been shown to be associated with the development of various tumors. Studies have demonstrated that the activation of TAS2R has various anticancer effects, such as increasing apoptosis and reducing cell proliferation and migration (Seo et al., 2017; Salvestrini et al., 2020; Zehentner et al., 2021). The expression level of most TAS2Rs is reduced in prostate cancer cell lines compared to cells with benign prostatic hyperplasia (Salvestrini et al., 2020). Several naturally bitter compounds from plants, such as bitter melon extract (BME) and Noscapine, have shown anti-cancer effects against a variety of cancer types. Recent studies have shown that Noscapine causes TAS2R14-dependent cell death induction and decreased prostate cancer cell viability (Martin et al., 2019). Momordica charantia is used as a functional food to prevent and treat human health-related problems. It was found that the extract of momordica balsam could block the cycle of prostate cancer cells in S phase, enhance the expression of Bax and induce PARP cleavage *in vitro* (Ru et al., 2011). Oral force-feeding of BME delayed progression to high-grade prostatic intraepithelial neoplasia in TRAMP (transgenic adenocarcinoma of mouse prostate) mice (31%), with prostate tissue showing about 51% reduction in expression of proliferating cell nuclear antigen (Ru et al., 2011). The results suggest that BME inhibits the progression of prostate cancer in TRAMP mice by interfering with cell cycle progression and proliferation. However, whether this process is mediated by bitter receptors is unknown and requires further exploration.

### 2.3 Other orphan G protein-coupled receptors

*GPR158* is an orphan receptor and a member of the glutamate family of GPCRs50. A microarray study showed that GPR158 was one of the upregulated genes in androgen-resistant ablative metastases compared to primary prostate tumors (Chandrashekar et al., 2006). Nitin et al. found that transient overexpression of GPR158 in prostate cancer cell lines significantly increased cell proliferation, and this effect was independent of AR function (Patel et al., 2015). Further studies found that GPR158 expression was stimulated by androgens and GPR158 stimulated AR expression, suggesting that it was possible to sensitize tumors to hypoandrogenic conditions through a positive feedback loop during androgen deprivation therapy (ADT) (Patel et al., 2015). In addition, GPR158 expression was found to correlate with neuroendocrine (NE) differentiation phenotypes and promote anchor-independent colony formation, suggesting a role for GPR158 in treatment progression and tumor formation (Patel et al., 2015).

Zhou et al. found in 2016 that transcription levels of *GPR160* in prostate cancer tissue samples and cell lines were significantly higher than in normal prostate tissue and cells (Zhou et al., 2016). After GPR160 knockdown, functional annotations of differentially expressed genes showed that cytokine activity, cell cycle phase, and mitosis were the most obvious functions of repressed-gene abundance, while programmed cell death, apoptosis, and the regulation of chemotaxis were significantly enriched by activated genes (Zhou et al., 2016). It is suggested that GPR160 has a potential role in the pathogenesis of prostate cancer, but the ligand of GPR160 has not been discovered at this time. In 2020, Gina et al. identified CARTp (cocaine- and amphetamine-regulated transcript peptide) as a ligand of GPR160, which plays an important role in neuropathic pain model (Yosten et al., 2020). The latest study directly involved prostatectomy tissue and analyzed GPR160 mRNA and protein levels for clinical relevance. The results showed that Gleason grade and stage were positively correlated with GPR160 mRNA level, and the presence of GPR160 was associated with cell migration (Guo et al., 2021). However, the specific mechanism of GPR160 in prostate cancer has not been revealed.

## 3 Chemokine receptors in prostate cancer

Chemokines are low molecular mass cytokines secreted by different types of cells that can make cells undergo chemotactic motion. Chemokine receptors are a type of GPCRs with seven transmembrane regions that can bind to chemokines and plays an important role in normal and abnormal physiological conditions (Lira and Furtado, 2012). Most chemokines bind to a variety of receptors with high affinity, inducing the activation of the second messenger to produce a strong signaling cascade, which leads to the chemotaxis and transport of target cells to fulfill a variety of biological functions. The occurrence and development of tumors are accompanied by many complex molecular events. In the tumor microenvironment, various chemokines or cytokines participate in inflammatory response and immune regulation, which play an important role in promoting tumor occurrence and progression, chemotherapy resistance and escaping immune surveillance (Nagarsheth et al., 2017). Chemokine receptors in prostate cancer have been summarized in Table 1.

**TABLE 1:** Chemokine receptors in prostate cancer.

Chemokine receptor	Ligand (s)	Signaling pathways	Biological functions in PCa	Therapeutic agents/Implications
CXCR4	CXCL12	PI3K/AKT, MAPK/ERK, JAK/STAT	Bone metastasis, EMT, migration, invasion, immune suppression	AMD3100 (plerixafor), balixafortide (in trials)
CXCR7	CXCL12, MIF	β-arrestin/MAPK, AKT	Cell survival, proliferation, drug resistance, decoy receptor function	CCX771 (inhibitor, preclinical)
CXCR1/CXCR2	CXCL8 (IL-8)	MAPK, NF-κB	Cell cycle progression, apoptosis regulation, influences AR expression, MDSC recruitment	AZ10397767 (CXCR2 antagonist, preclinical)
CXCR6	CXCL16	JAK/STAT, PI3K	Facilitation of metastasis via CAF differentiation and EMT induction	Under exploration

### 3.1 CXCR4

Among more than 20 chemokine receptors that have been found, chemokine receptor type 4 (CXCR4) is most closely associated with tumors, and is also an auxiliary receptor for HIV infection (Vandercappellen et al., 2008). CXCR4 expression is significantly higher than that in normal tissues in various tumors and different stages of tumors. Studies have confirmed that CXCR4 is related to the proliferation, adhesion, invasion and metastasis of tumor cells, and plays an important role in tumor progression (Alsayed et al., 2022). Molecular analysis showed that 44.0% of the samples isolated from circulating tumor cells (CTCs) of 48 mPCa patients were positive for CXCR4. The expression of the CXCR4 protein was linked to worse progression - free survival (PFS) (Roumeliotou et al., 2024).

CXCL12/CXCR4 pathway biological axis is an important regulator of the spread of prostate cancer. Inhibition of CXCL12/CXCR4 pathway has been shown to reduce the incidence of bone metastasis in prostate cancer (Miwa et al., 2005; Sun et al., 2025). CXCR4 can form homodimers or heterodimerize with other GPCRs to amplify or reduce the signaling capacity of each receptor. The influence of CXCR4 and its close collaborator CXCR7 on different physiological and pathophysiological processes has been studied extensively over the past decades. When activated by their shared ligand-CXCL12, both chemokine receptors induce various cellular signaling pathways and/or clear CXCL12 from the extracellular environment (Huynh et al., 2020).

Studies have confirmed that CXCL12 chemokines and their receptors CXCR4 and CXCR7 may be involved in the initiation and EMT process of prostate cancer metastasis (Parol-Kulczyk et al., 2022). Obesity is a prognostic risk factor for prostate cancer progression. In a myc-induced obesity-driven mouse model of prostate cancer, immunofluorescence staining of ventral prostate tissue shows high levels of CXCL12 in stromal compartments and high staining of CXCR4 and CXCR7 in tumor epithelial compartments (Saha et al., 2017). CXCL12 treatment stimulates the migration and invasion of prostate cancer cells, which are inhibited by the CXCR4 antagonist AMD3100 and the combination of CXCR4 or CXCR7, suggesting that CXCL12-CXCR4/CXCR7 signaling axes can be potential targets for PCa intervention (Saha et al., 2017). Data indicated a prevalent immunosuppressive state among CD8 T cells, accompanied by variations in their abundance. The CXCL12/CXCR4 axis played a pivotal role in this process, functioning as a critical mediator. Macrophages emerged as key regulators, utilizing the CXCL12/CXCR4 axis to recruit CD8^+^ effector T cells and regulatory T cells (Tregs) into the tumor microenvironment (TME) (Zhang Y. et al., 2025). Some Chinese herbs can also affect the progression of PCa by targeting the CXCL12/CXCR4 pathway. As the active component of the Yishen Tongluo Jiedu recipe, quercetin inhibited the development of PCa through the Akt1-related CXCL12/CXCR4 pathway (Ning et al., 2024).

In addition to close collaboration with CXCR7, using biophysical and biochemical methods, CXCR4 is found to form induced heterodimers with the cannabinoid receptor 2 (CB2) in human breast and prostate cancer cells (Coke et al., 2016). Agonist-dependent activation of CXCR4 and CB2 results in reduced expression of phosphorylated ERK1/2 mediated by CXCR4 and ultimately reduced cancer cell functions such as calcium mobilization and cell chemotaxis (Coke et al., 2016). During inflammation and tissue regeneration, CXCL12 forms a heterocomplex with HMGB1 and acts via CXCR4, which is crucial for cell migration and invasion. Study shows that cancer cells’ responsiveness to the heterocomplex, leading to increased migration and invasiveness, depends on HMGB1 remaining in its reduced isoform. The study also proposes disrupting the heterocomplex as a potential therapeutic target to inhibit cancer invasion and metastasis, emphasizing the important roles of CXCL12 and CXCR4 in this process (Pirani et al., 2024).

### 3.2 CXCR7

CXCR4 has been considered to be the only receptor for CXCL12. In recent years, with the deepening of studies on chemokines and their receptors, Balabanian et al. confirmed that CXCR7 has a high affinity with CXCL12, and pointed out that CXCR7 is the second receptor of CXCL12 (Balabanian et al., 2005). Miao et al. (2007) detected CXCR7 expression in most common human tumors such as lung cancer, breast cancer, cervical cancer, kidney cancer and rhabdomyosarcoma through immunohistochemistry, which is widely involved in multiple biological functions such as inflammatory response, immune response and angiogenesis (Shi et al., 2020). Although both can bind to CXCL12, the specific roles of CXCR4 and CXCR7 are different. For example, during the homing process of renal progenitor cells induced by CXCL12, CXCR4 mainly mediated the chemotaxis and migration of renal progenitor cells, while CXCR7 mainly promoted the survival and their adhesion to endothelial cells (Mazzinghi et al., 2008). CXCR7 cannot cause Ca2+ migration after activated by CXCL12, suggesting that CXCR7-mediated signaling pathway is different from that mediated by typical chemokines (Burns et al., 2006). In addition, CXCR7 could form heterodimers with other chemokine receptors, or exist as a non-signaling inducer (Sierro et al., 2007). Kalatskaya et al. (2009) found that although AMD3100, a selective small molecule inhibitor of CXCR4, could also bind to CXCR7 receptors, However, it was an allosteric agonist of CXCR7. Therefore, whether CXCR4/CXCL12 and CXCR7/CXCL12 signaling pathways are independent or synergistic, the specific regulatory factors of their signaling pathways still need to be further studied and discussed.

A joint clinical study by Prof. Doutor Fernando Fonseca Hospital and Portugal and Portuguese Oncology Institute of Porto collected between 2000 and 2005 Specimens from 234 patients who underwent radical retropubic prostatectomy. Analysis showed that patients with higher CXCR7 expression were more likely to have disease recurrence in relation to disease-free survival (DFS), suggesting that CXCR7 was an independent predictor of poor DFS after radical prostatectomy (Bargao Santos et al., 2020). Yang et al. confirmed the high expression level of CXCR7 in PCa tissues and evaluated the relationship between CXCR7 expression and clinicopathological parameters. Results showed that CXCR7 is a valuable prognostic biomarker for PCa patients and may promote the invasive progression of PCa by enhancing the proliferation and migration of tumor cells (Yang et al., 2018). CXCR7 has been shown to inhibit the development and progression of tumors through multiple biological processes, either through receptor-ligand binding or through non-classical pathways, i.e., ligand-independent activation. Knockdown of CXCR7 in CaP cells induced a variety of antitumor effects, including reduced cell proliferation, cell cycle stasis in the G1 phase, and reduced expression of proteins involved in the progression of G1 to S phases (Singh and Lokeshwar, 2011). In contrast, the addition of CXCR7 ligands SDF-1a and CXCL11 to CaP cells did not affect cell proliferation (Singh and Lokeshwar, 2011). Meanwhile, the loss of CXCR7 expression also resulted in the serious impairment of EGFR signaling and the onset of cell senescence (Hoy et al., 2017). In conclusion, the proliferation, survival and clonogenesis potential of prostate cancer depend on CXCR7. However, the functional role of CXCR7 is complex and may be context-dependent. For instance, downregulation of CXCR7 protein has been shown to increase migration in some settings, supporting a role for CXCR7 as a decoy receptor that sequesters CXCL12 and thereby counteracts CXCL12/CXCR4-mediated migration in prostate cancer (Yu et al., 2020). This contrasts with other studies highlighting its direct pro-tumorigenic signaling functions, underscoring the need to fully elucidate the contexts that determine its dual nature.

Although ADT is an effective treatment for metastatic prostate cancer, incurable castration-resistant prostate cancer (CRPC) inevitably develops (Rafiei et al., 2019). Enzalumide is a new generation of AR antagonist, and its drug resistance leads to prostate cancer becoming a more refractory tumor (Li et al., 2019). CXCR7 is one of the most upregulated genes in Enzalumide resistant cells (Li et al., 2019). Yong et al. conducted a clinical study of patients diagnosed with CRPC between January 2015 and December 2019. Prostate biopsy tissues were divided into CXCR7 positive group and CXCR7 negative group according to CXCR7 expression. Patients with lower CXCR7 expression showed a better PSA response to Enzalumide treatment (Luo et al., 2021). Further stratified analysis of all CXCR7-positive patients showed that those with higher CXCR7 expression had a worse prognosis (Luo et al., 2021). *In vitro* studies, AR directly inhibited CXCR7 by binding to an enhancer at 110 kb downstream of the CXCR7 gene, and its expression was restored after androgen deprivation (Li et al., 2019). These results suggest that CXCR7 can be used as a biomarker of drug-resistant disease in prostate cancer patients, and that disrupting CXCR7 signaling may be an effective strategy to overcome drug resistance.

Macrophage migration inhibitor (MIF) has been identified as a ligand of CXCR7, which induced cell cycle gene expression through activation of the AKT signaling pathway (Rafiei et al., 2019). Studies have shown that the MIF/CXCR7/AKT pathway drives CRPC growth and metastasis independently of the CXCL12/CXCR4 axis (Rafiei et al., 2019). Different from CXCR4, CXCR7 can also affect the occurrence and progression of tumors in CRPC through a ligand-independent but β-arrestin 2-dependent mechanism. CXCR7 was found to influence MAPK/ERK signaling via β-arrestin 2, and β-arrestin 2 consumption increased proliferation/colony formation and significantly increased Src activation, EGFR phosphorylation at Tyr-1110, and ERK1/2 phosphorylation/activation (Kallifatidis et al., 2016; Li et al., 2019). Levels of p-EGFR (Y1068), p-AKT (T308) and VEGFR2 in prostate cancer cells were reduced after Enzalumide combined with CCX771 (a CXCR7 inhibitor) compared with single drug therapy (Luo et al., 2018). In addition, migration was significantly reduced after combination therapy, and importantly, a similar reduction was observed in levels of secreted VEGF (Luo et al., 2018). It is suggested that the combination of Enzalumide and CXCR7 may inhibit CRPC growth and possibly prevent metastasis, in part due to the reduction of pro-angiogenic signaling and macroangiogenesis.

Currently, a number of studies have shown that CXCR7 can be used as a therapeutic target for prostate cancer, and the progression and metastasis of prostate cancer can be inhibited by targeting CXCR7 (Zheng et al., 2013; Mir et al., 2016; Bai et al., 2019; Zhang Y. et al., 2025). However, compared with CXCR4 and CXCL12 with many modulators entering clinical development (Pernas et al., 2018; Ghobrial et al., 2020; Wang et al., 2020), the research of CXCR7 regulators is still lagging behind. Therefore, more comprehensive and in-depth clinical studies are needed to evaluate the clinical application value of CXCR7 in prostate cancer.

### 3.3 Other CXCRs

In 2006, Tobias et al. detected the expression of CXCL1, CXCL3, CXCL5 and CXCL6 in prostate cancer cell lines DU-145 and PC-3, but not in LNCaP (Engl et al., 2006). Further studies found that inhibition of CXCL8 and its receptor chemokines CXCR1 and CXCR2 promote G1 cell cycle arrest and apoptosis, suggesting that CXCR1 and CXCR2 inhibit tumor progression in prostate cancer by influencing cell proliferation. Meanwhile, by enhancing CXCR2 expression, AR expression was antagonistically regulated (Dahal et al., 2023). In androgen-independent prostate cancer (AIPC), co-administration of CXCR2 selective antagonist AZ10397767 attenuated oxaliplatin induced NF-κB activation, thus increasing oxaliplatin cytotoxicity and enhancing oxaliplatin induced apoptosis of AIPC cells (Wilson et al., 2008). In terms of tumor metastasis, CXCR6 signaling stimulated the transformation of mesenchymal stem cells into cancer-associated fibroblasts, which secreted stroma-derived factor 1, also known as CXCL12. CXCL12 expressed by cancer-associated fibroblasts then banded to CXCR4 on tumor cells and induced epithelial-mesenchymal transformation, ultimately promoting metastasis to secondary tumor sites (Jung et al., 2013). However, the research on the function of the above chemokine receptors is still weak and needs further exploration and discovery.

Beyond the receptors discussed above, the CXCL8-CXCR1/2 axis is also implicated in PCa progression. Notably, CXCR2 serves as a key receptor for multiple chemokines (e.g., CXCL1, CXCL2, CXCL8) and is critically involved in the recruitment of myeloid-derived suppressor cells (MDSCs), a potent population of immunosuppressive cells, into the tumor microenvironment (Liu et al., 2024). The infiltration of MDSCs facilitates immune evasion and is associated with therapy resistance, highlighting another layer of GPCR-mediated immunomodulation in PCa (Lasser et al., 2024).

## 4 Hormone-responsive GPCRs in prostate cancer

The regulation of AR on prostate cancer, and the blocking of AR signaling pathway by AR antagonists and sterogenic enzyme inhibitors have been widely studied. In addition to the key roles of androgens and AR in the regulation of prostate cancer pathogenesis, a variety of hormone-responsive GPCRs are also involved in the occurrence and development of prostate cancer, so they may serve as potential alternative drug targets for prostate cancer intervention. These proteins include the gonadotropin releasing hormone receptor (GnRHR), luteinizing hormone receptor (LHR), and follicle-stimulating hormone receptor (FSHR), which are key components of the hypothalamic-pituitary-gonadal (HPG) axis, the relaxin receptor, ghrelin receptor (GHRP), and basal peptidine receptor (also known as GPR54).

### 4.1 GnRHR

Pituitary GnRHR is a key component of the HPG axis and plays a key role in regulating the synthesis and secretion of gonadotropin. It is known that the GnRHR is expressed in different types of cancer, including CRPC, and mediates the anti-proliferative effects of GnRH analogues (Montagnani Marelli et al., 2015). Adenocarcinoma sublines of Dunning rats with different grades of prostate tumor showed significantly increased GnRHR mRNA expression levels compared with normal rats, while GnRHR mRNA levels showed no difference between different tumor grades (Tieva et al., 2005). Studies have shown that GnRH-II has specific and significant anti-proliferation effects on prostate cancer cells. This antitumor effect was mediated by the activation of type I (but not type II) GnRHR and its associated cAMP intracellular signaling pathway (Montagnani Marelli et al., 2009). GnRH analogues can also play a local role in the prostate gland by triggering PCa cell apoptosis through activation of GnRHR (Sanchez et al., 2021). GnRHR plays a role in PCa by changing its distribution in the cell, reducing its expression in the cell membrane, and remaining isolated in the endoplasmic reticulum (Sanchez et al., 2021).

Targeted GnRHR has been shown to be an effective treatment for prostate cancer and offers potential for combination treatment. GV1001, a fragment of human telomerase reverse transcriptase, with high binding affinity with the prediction of binding to GnRHR, was a potential novel GnRHR ligand capable of inhibiting PCa metastasis via the Gαs/cAMP pathway (Kim et al., 2021). Degarelix is a gonadotropin-releasing hormone GnRHR antagonist used in prostate cancer patients requiring androgen deprivation therapy. It was found that Degarelix has a direct effect on prostate cell growth through apoptosis, and may also be effective against BPH (Sakai et al., 2015). GnRHR can also bind to gemcitabine, producing a molecule that is metabolically superior to the gemcitabine molecule (GSG) and inhibits tumor progression in CRPC animal models (Karampelas et al., 2014).

### 4.2 LHR

Most drug discovery attempts targeting the HPG axis for prostate cancer have focused on GnRHR. However, the available treatments for LHR or FSHR are limited. Luteinizing hormone regulates testosterone synthesis in men and ovulation and luteal development in women. The receptor that mediates its action, LHR, is a glycosylated GPCRs-like protein. LHR is known to signal primarily through the Gs-cAMP-PKA pathway and, to a lesser extent, the Gq-PLC-Ca2+ signaling pathway to confer physiological function (Gudermann et al., 1992).

Expression of LH and FSH receptors was detected in glandular epithelial cells and stroma of prostate tissue samples. The expression of both receptors was higher in glandular epithelial cells than in the interstitium of all prostate regions. In glandular epithelial cells, LHR and FSHR are expressed laterally less than in other regions, and there is no difference between the dorsal and ventral regions. However, no changes in LHR and FSHR expression were found in the interstitium (Ponglowhapan et al., 2012). These findings indicate that changes in the expression levels of LHR and FSHR observed in their regions and tissue layers suggest a potential role for gonadotropins LH and FSH in the physiological regulation of the prostate. MRNA and protein expression of LH and LHR are also found in LNCaP and PCA cells. We found that LH-mediated LHR activation significantly upregulated the expression of genes and enzymes required for steroid production and increased steroid production in PCA cells (Pinski et al., 2011). Treatment with LHR-siRNA can prevent LH-mediated proliferation and androgen synthesis of prostate cancer cells, and downregulate the expressions of AR, PSA, PKA, ERK1/2, PI3K, AKT2 and HER2 (Xiong et al., 2015).

In conclusion, LHR may regulate multiple signaling pathways in prostate cancer. Furthermore, LH-mediated LHR activation not only upregulates steroidogenic enzymes but may also promote intracrine androgen synthesis, providing a local source of androgens to activate the AR signaling axis even under castrate conditions, thereby contributing to CRPC development. However, the specific function and mechanism need to be further explored.

### 4.3 FSHR

As a key regulator of mammalian reproduction, FSH is thought to regulate the occurrence of gametophytes and steroid production, as well as subsequent development and growth of sexual organs and the onset of puberty. FHSR signals mainly through the Gs-cAMP-PKA cascade to upregulate the expression of target genes responsible for steroid production in ovarian granulosa cells and testicular sertoli cells (Hunzicker-Dunn et al., 2012). Other signaling pathways, such as protein kinase B, glucocorticoid-induced kinase, and p38 MAPK, can be activated by FSHR in a PKA-independent manner (Gonzalez-Robayna et al., 2000).

FSHR expression was generally higher in prostate cancer samples than in normal prostate and BPH samples. PC3 cells expressed FSHR, while LNCaP cells were negative for FSHR (Mariani et al., 2006). In prostate cancer metastases, the density of FSHR-positive blood vessels in metastases was about 3 times higher than in the tumor interior, suggesting that FSHR may be significantly associated with, and potentially actively contributes to, prostate cancer metastasis (Sakai et al., 2015). This striking clinical correlation positions FSHR as a potential key player in the metastatic process, meriting further investigation into its mechanistic role. Chemical castration of prostate cancer can be achieved by GnRH agonists or antagonists. Although both initially inhibit LH and FSH, FSH levels rebound during agonist treatment. Studies have evaluated the effect of human recombinant FSH on tumor growth after gonadotropin inhibition with Degarelix, a GnRH antagonist. It was found that FSH supplementation reversed induced tumor inhibition, both prophylactically (Degarelix and FSH therapy were initiated after cell inoculation) and therapeutically (therapy was initiated 3 weeks after cell inoculation) (Oduwole et al., 2021). Therefore, FSHR and its ligands may play an important role in the regulation of growth in hormone-refractory prostate cancer, and the rebound of FSHR during treatment may be a vital reason for the further progression of the disease.

### 4.4 Other hormone-responsive GPCRs

Relaxin, a member of the insulin-like family, is a multifunctional regulator of a variety of physiological processes, especially well-known functions related to the female reproductive system during pregnancy (Hall, 1947), whose homologous receptor RXFP is a member of GPCRs. MRNA expression of relaxin and RXFP was confirmed in prostate and testis of normal mice (Samuel et al., 2003). RXFP1 is expressed in both AR positive and negative prostate cancer and prostate cancer cell lines. Intra tumor injection of RXFP1 siRNA resulted in downregulation of RXFP1 receptor expression and a significant reduction in tumor growth. Global transcription profiling revealed that RXFP1 siRNA significantly altered the expression of tumor-promoting genes (Feng et al., 2010). These studies suggest that relaxin antibodies are important factors in the growth and proliferation of prostate cancer.

Ghrelin is a novel growth hormone-releasing peptide originally identified as an endogenous ligand of the GHSR in rat stomachs. Ghrelin is involved in the regulation of GH release, but recently it has been suggested that ghrelin may have other roles, including effects on appetite, carbohydrate metabolism, heart, kidney, pancreas, gonads, and cell proliferation (Yanagi et al., 2018). GHSR subtypes 1a and 1b and auxin releasing peptide mRNA were expressed in ALVA-41, LNCaP, DU145 and PC3 prostate cancer cell lines. Immunohistochemical staining of GHSR subtype 1a and auxin releasing peptides was also positive in the four cell lines (Jeffery et al., 2002). Studies have shown that GHRP antagonists could inhibit the growth of human prostate cancer DU145 cells (Lawnicka et al., 2012), but the specific function and mechanism of GHRP remain unclear.

GPR54 is one of a GPCRs, which previously an orphan receptor and later identified as a receptor for Kisspeptin (KISS1) (Makri et al., 2008). Recent functional studies of suggested that this receptor played an important role in regulating sex hormones, including GnRH (Li et al., 2022). Downregulation of the KISS1-GPR54 system was detected in advanced prostate cancer (Xoxakos et al., 2020). KISS1-GPR54 has been identified as an important intervention target in breast cancer migration (Wang et al., 2017), but its potential as a diagnostic, risk assessment, and therapeutic target for aggressive tumors in prostate cancer remains to be explored.

## 5 Conclusion

Although GPCR is the largest family of cell surface receptors and plays an important role in signal transduction, in clinical practice, only a small number of anticancer compounds play their role by interfering with GPCR-mediated signaling pathways. The role of GPCR and its ligands in prostate cancer progression is complex, however, more and more recent evidence suggests that these molecules are associated with tumor progression.

**FIGURE 3:**
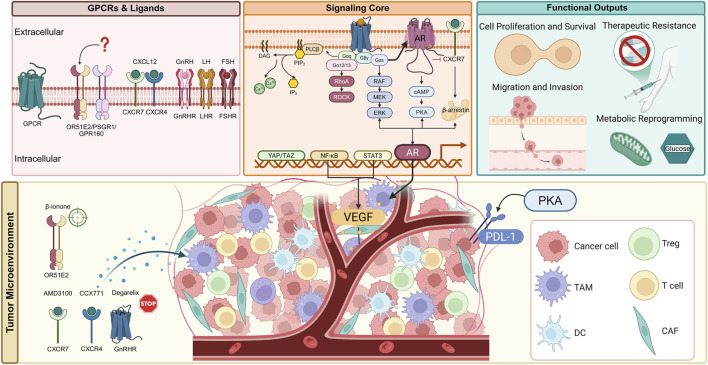
Multifaceted roles of GPCR signaling networks in prostate cancer pathogenesis and therapeutic targeting. This schematic integrates established and emerging concepts, illustrating how three GPCR subfamilies-orphan receptors, chemokine receptors, and hormone-responsive receptors-orchestrate prostate cancer progression. The diagram highlights the complex intracellular signaling network, profound crosstalk with the androgen receptor (AR) axis, extensive remodeling of the tumor microenvironment (TME), and the ensuing hallmarks of cancer. Key therapeutic strategies and agents targeting these pathways are indicated, showcasing the potential for precision oncology in advanced prostate cancer. AMD3100: CXCR4 antagonist; CCX771: CXCR7 inhibitor; Degarelix: GnRH receptor antagonist; β-ionone: OR51E2 agonist.

We propose a “GPCR–AR–TME” framework, linking the three GPCR subfamilies reviewed here to the hallmarks of PCa (Figure 3). Orphan receptors often drive proliferation; chemokine receptors are pivotal for metastasis, immune evasion, and therapy resistance; and hormone-responsive receptors can bypass classical androgen signaling, in part by stimulating intracrine androgen synthesis. This framework underscores how GPCRs serve as central nodes integrating signals from the tumor microenvironment, AR pathway, and intrinsic oncogenic drivers. Our synthesized framework is further supported by emerging insights. For instance, GPCR signaling can activate the Hippo-YAP/TAZ pathway, which cooperates with AR and other transcription factors to drive aggressive tumor phenotypes (Khalilimeybodi et al., 2023). The crosstalk between cancer cells and the TME is bidirectional; while GPCRs in cancer cells promote the recruitment of immunosuppressive cells and angiogenesis, signals from the TME continuously activate tumor GPCRs, creating a feed-forward loop (Wu et al., 2023; Williams et al., 2024; Li et al., 2025). Moreover, GPCR signaling can upregulate PD-L1 expression on tumor cells, directly linking it to immune checkpoint regulation (Khan et al., 2023; Topalian et al., 2023).

Therefore, GPCR and its downstream activation effectors are very important potential drug targets in the prevention and treatment of prostate tumors. In the future research field, the exploration of the network diagram of GPCR-mediated signaling pathway will further promote the understanding of this receptor, and provide scientists with a new method for the study of anti-prostate cancer drugs. In addition, ongoing studies of orphan receptors in GPCR may lead to the development of new anti-tumor targets in the future.

This review was based on a systematic search of PubMed, Web of Science, and Google Scholar databases. Key search terms included: “G protein-coupled receptor”, “GPCR”, “prostate cancer”, “prostate carcinoma”, “castration-resistant”, “orphan receptor”, “chemokine receptor”, “CXCR4″, “CXCR7″, “hormone receptor”, “GnRHR”, “therapeutic target”, and “drug discovery”. The search covered literature published from 1990 up to March 2025. The selection prioritized original research articles, high-impact reviews, and clinical studies that provided mechanistic insights or therapeutic perspectives on GPCRs in prostate cancer.
